# Real-world implementation of the National Early Warning Score-2 in an acute respiratory unit

**DOI:** 10.1136/bmjresp-2023-002095

**Published:** 2024-01-31

**Authors:** Sherif Gonem, Joseph Lemberger, Abdulla Baguneid, Steve Briggs, Tricia M McKeever, Dominick Shaw

**Affiliations:** 1 Department of Respiratory Medicine, Nottingham University Hospitals NHS Trust, Nottingham, UK; 2 NIHR Nottingham Biomedical Research Centre, University of Nottingham, Nottingham, UK; 3 Department of Oncology, Nottingham University Hospitals NHS Trust, Nottingham, UK; 4 Digital and Information, Nottingham University Hospitals NHS Trust, Nottingham, UK; 5 Lifespan and Population Health, University of Nottingham, Nottingham, UK

**Keywords:** Critical Care

## Abstract

**Introduction:**

The National Early Warning Score-2 (NEWS-2) is used to detect deteriorating patients in hospital settings. We aimed to understand how NEWS-2 functions in the real-life setting of an acute respiratory unit.

**Methods:**

Clinical observations data were extracted for adult patients (age ≥18 years), admitted under the care of respiratory medicine services from July to December 2019, who had at least one recorded task relating to clinical deterioration. The timing and nature of urgent out-of-hours medical reviews (escalations) were extracted through manual review of the case notes.

**Results:**

The data set comprised 765 admission episodes (48.9% women) with a mean (SD) age of 69.3 (14.8). 8971 out of 35 991 out-of-hours observation sets (24.9%) had a NEWS-2 ≥5, and 586 of these (6.5%) led to an escalation. Out of 687 escalations, 101 (14.7%) were associated with observation sets with NEWS-2<5. Rising oxygen requirement and extreme values of individual observations were associated with an increased risk of escalation. 57.6% of escalations resulted in a change in treatment. Inpatient mortality was higher in patients who were escalated at least once, compared with those who were not escalated.

**Conclusions:**

Most observation sets with NEWS-2 scores ≥5 did not lead to a medical escalation in an acute respiratory setting out-of-hours, but more than half of escalations resulted in a change in treatment. Rising oxygen requirement is a key indicator of respiratory patient acuity which appears to influence the decision to request urgent out-of-hours medical reviews.

**WHAT IS ALREADY KNOWN ON THIS TOPIC**

- The National Early Warning Score-2 (NEWS-2) is a physiological scoring system that is mandated for use in acute hospitals by National Health Service England, but it is not known how NEWS-2 functions within real-life clinical settings, including how it interacts with clinical decision-makers to lead to urgent out-of-hours medical reviews, and how often high NEWS-2 alerts lead to significant changes in patient treatment.

**WHAT THIS STUDY ADDS**

- High NEWS-2 scores occur commonly on acute respiratory wards, and the majority of these do not lead to medical escalations, but more than half of escalations that do occur lead to a change in treatment. Rising oxygen requirement and extreme values of individual observations are factors which appear to be taken into account by clinical decision-makers when deciding whether to request a medical escalation.

**HOW THIS STUDY MIGHT AFFECT RESEARCH, PRACTICE OR POLICY**

- Further research is needed to determine whether the failure to escalate patients with high NEWS-2 scores leads to adverse outcomes, and whether changes to the NEWS-2 scoring system may help to mitigate this. The addition of rising oxygen requirement as a separate contributor to the score merits further investigation.

## Introduction

Early warning scores are used in hospital settings as screening tools for acute clinical deterioration. Their purpose is to enable the early recognition and treatment of time-critical conditions such as sepsis. They are often used as part of a rapid response system, in which the detection of patient deterioration triggers an urgent review by a doctor or specialist nurse. The National Early Warning Score (NEWS) was introduced in 2012, and an updated version (NEWS-2) was published in 2017,^1^ with the inclusion of a separate oxygen saturation scale for patients with chronic respiratory disease at risk of hypercapnic respiratory failure.

The key threshold for triggering a medical escalation recommended in current guidelines is a NEWS-2 of ≥5. There has been concern that this could cause an unmanageable number of alerts to the on-call medical team if it was implemented without any form of filtering.^2–4^ Hospitals may mitigate this by allowing ward nurses at a certain level of seniority to de-escalate high NEWS-2 alerts according to their clinical judgement, for instance if a treatment plan is already in place. Conversely, ward nurses can also choose to escalate patients who have a concerning physiological deterioration, even if the NEWS-2 does not reach the threshold of ≥5. Nottingham University Hospitals NHS Trust (NUH) has a mature rapid response system which is known locally as Hospital 24. This comprises a team of experienced nurses (known as Hospital 24 coordinators) who have access to real-time clinical observations and NEWS-2 scores for all patients in the hospital, through an electronic task management system (Nervecentre). The Hospital 24 coordinators respond to high NEWS-2 alerts and direct escalations from ward nurses by sending tasks to the on-call medical team to review patients who appear to be deteriorating. These tasks are assigned and tracked using the same Nervecentre system.

Several retrospective studies have evaluated the performance of NEWS and NEWS-2 in predicting mortality, cardiac arrest and intensive care unit admission.^5–8^ However, we are not aware of any previous studies which have investigated how NEWS-2 leads to activation of the rapid response system in a real-life setting. This information is needed to inform workforce planning, and to determine whether any changes need to be made to NEWS-2 to reduce unnecessary medical reviews and prevent failure-to-rescue events. This study focused on the use of NEWS-2 outside of usual office hours (ie, during evenings and weekends), because our rapid response system is only operational at these times. During usual office hours (09:00 to 17:00, Monday to Friday) patients are reviewed on a daily basis as a matter of routine, and deteriorating patients are escalated directly to the ward doctors.

The aims of this observational study were, in an acute respiratory inpatient population:

1. To establish what proportion of high NEWS-2 scores lead to an urgent medical review in the out-of-hours setting, and to determine what factors are associated with the decision to escalate or de-escalate a given NEWS-2 alert.
2. To understand how often NEWS-2 fails to detect deteriorating patients in hospital, by establishing the proportion of urgent out-of-hours medical reviews, relating to an acute physiological disturbance, which do not reach the NEWS-2 escalation threshold of ≥5.
3. To establish what proportion of urgent out-of-hours medical reviews lead to a significant change in treatment, and to determine what factors are associated with medical reviews that result in a change in treatment.
4. To determine the inpatient mortality rate for patients who received an urgent out-of-hours medical review compared with those who did not.

## Methods

### Study population

Adult patients (age ≥18 years) admitted under the care of respiratory medicine services at NUH between 1 July 2019 and 31 December 2019 inclusive, who had at least one patient review task relating to clinical deterioration recorded on the Nervecentre system. These were identified using the category labels ‘Acutely unwell / urgent response’, ‘Clinical review / management (Red)’, ‘Early warning score>4’, ‘High NEWS: Inform registrar’ and ‘Reg alert’.

### Data source

We extracted clinical observations data from an electronic task management system (Nervecentre) which has been in use at NUH since 2015. These comprised date and time-stamped records of heart rate, blood pressure, respiratory rate, temperature, oxygen saturation, inspired oxygen concentration or flow rate and level of consciousness, recorded using the five-point ACVPU scale (Alert, Confused, responds to Voice, responds to Pain, Unresponsive).

### Inspired oxygen categories

Inspired oxygen is treated as a binary variable in NEWS-2, with zero points for no oxygen and two points for receiving any supplemental oxygen. In order to investigate the significance of inspired oxygen flow rate (L/min) or concentration (fractional inspired oxygen (FiO_2_), %), we defined supplemental oxygen categories as follows:

None

Low: Flow rate 0.5–2.5 L/min or FiO_2_ 22–24%.

Low-moderate: Flow rate 3–4 L/min or FiO_2_ 25–28%.

Moderate: Flow rate 5–9 L/min or FiO_2_ 29–35%.

High: Flow rate 10–14 L/min or FiO_2_ 36–50%.

Very high: Flow rate ≥15 L/min or FiO_2_≥51%.

### Calculation of NEWS-2

NEWS-2 was calculated as per current guidelines.^1^ Oxygen saturation Scale 2, with target saturations of 88–92%, was used for observation sets labelled as ‘Chronic Respiratory Disease’. This included the over-oxygenation penalty, applied if oxygen saturations were ≥93% and the patient was receiving supplemental oxygen. Otherwise, Scale 1, with target saturations of 94–98%, was used. NEWS-2 was only calculated for complete observation sets.

### Review of escalations

We defined escalations as urgent medical reviews that occurred outside the hours of 09:00 to 17:00, Monday to Friday and were related to a raised NEWS-2 or an abnormality in one or more parameters of NEWS-2. We focused on out-of-hours reviews as this is when the Hospital 24 rapid response system is operational. The information held in the task management database did not allow the timing of escalations to be reliably retrieved in an automated fashion. Therefore, this was carried out through manual review of the case notes for each admission episode within the data set. Each escalation was labelled according to whether it resulted in a change in treatment. Examples of changes in treatment included giving a fluid bolus, or commencing antibiotic or diuretic therapy. If there was no change, the reason for this was recorded as ‘Patient clinically stable’, ‘Plan already in place’ or ‘No further treatment to offer’. Case note annotation was carried out by one of three investigators (SG, JL and AB). JL and AB met with the lead investigator (SG) to ensure a consistent approach to case annotation.

### Data analysis

Statistical analysis was performed using SPSS Statistics V.28 (IBM, Armonk, New York, USA). A p value of <0.05 was taken as the threshold for statistical significance. Incomplete observation sets and associated events were excluded from the analysis.

Risk ratios and 95% CI were calculated for the risk of escalation with rising versus stable inspired oxygen, and with and without at least one NEWS-2 parameter scoring the maximum number of points (three). Rising inspired oxygen was defined as a higher inspired oxygen category on the current observation set compared with the previous one. The χ^2^ test was used to compare the proportion of escalations that resulted in a change in treatment according to normal or abnormal NEWS-2 parameters, and to compare the inpatient mortality rates for patients who were escalated at least once compared with those who were not. A logistic regression model was constructed to determine whether escalation was a significant predictor of mortality, independently of the average NEWS-2 score.

### Patient and public involvement

Written feedback on the study design was received from a panel of patients and members of the public prior to its initiation. The panel was supportive of the study aims and methods.

## Results

### Data set characteristics

The extracted data set comprised 765 admission episodes (48.9% women) with a mean (SD) age of 69.3 (14.8), and a hospital mortality rate of 14.6%. Manual review of the case notes revealed 691 escalation events, of which 687 were associated with complete observation sets and were included in the analysis. 328 out of 765 admission episodes (42.9%) had at least one escalation event, while 437 (57.1%) did not.

**Figure 1:**
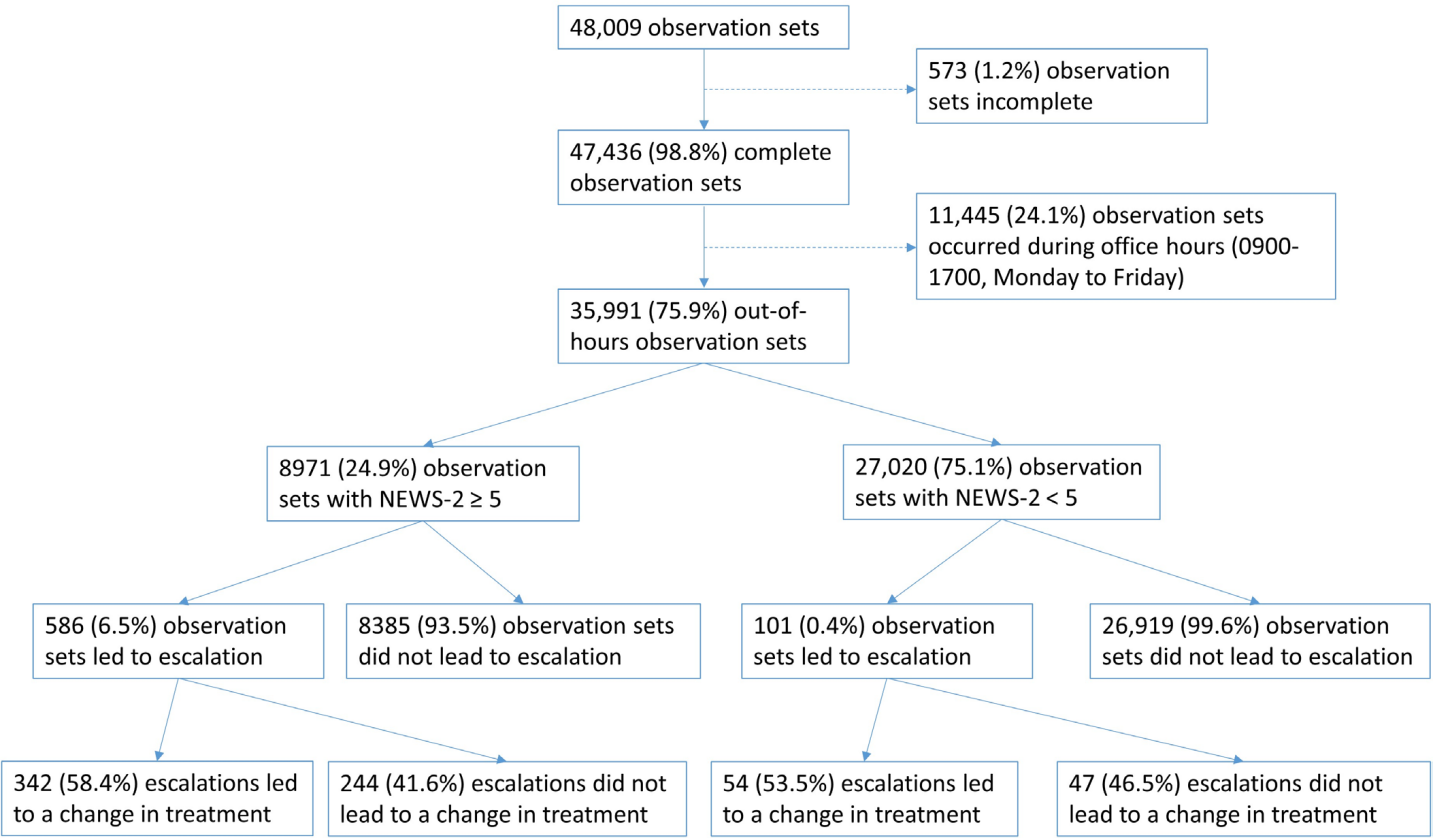
Flow chart of analysed observation sets and escalations. NEWS-2, National Early Warning Score-2.

The data set contained 48 009 clinical observation sets, of which 47 436 (98.8%) were complete. 11 445 of these were recorded during normal office hours of 09:00 to 17:00, Monday to Friday and were excluded from the analysis (figure 1). The remaining 35 991 complete observation sets were recorded outside these hours, and were included in the analysis. Figure 1 shows the number of observation sets with NEWS-2≥5 or <5, the number which led to an escalation and of these, the number which resulted in a change in treatment. 8971 out of 35 991 observation sets (24.9%) had a NEWS-2≥5, and 586 of these (6.5%) led to an escalation. Of the 35 991 observation sets, 27 020 (75.1%) had a NEWS-2<5 and 101 of these (0.4%) led to an escalation. Out of 687 escalations, 586 (85.3%) were associated with a NEWS-2≥5 and 101 (14.7%) were associated with a NEWS-2<5. Overall, 396 out of 687 (57.6%) escalations resulted in a change in treatment.

### Factors associated with escalations

Table 1 shows the risk of escalation for any given inspired oxygen category, depending on whether it was rising or stable. In each case, the risk of escalation was greater with rising compared with stable inspired oxygen. Table 2 shows the risk of escalation for any given NEWS-2 score with and without at least one parameter scoring the maximum number of points (three). This shows that one or more highly abnormal observations (scoring 3) appear to carry a greater risk of escalation than multiple mildly abnormal observations, particularly when the total NEWS-2 is ≤5. The risk ratio was significantly greater than one for NEWS-2 scores of 3, 4, 5 and 7.

**Table 1:** Risk of escalation according to rising or stable inspired oxygen

Inspired oxygen category	Stable inspired oxygen*	Rising inspired oxygen*	Risk ratio (95% CI)†
**None** Flow rate (L/min): 0 FiO_2_ (%): 21	135/14 400 (0.9)	–	–
**Low** Flow rate (L/min): 0.5–2.5 FiO_2_ (%): 22–24	182/11 043 (1.6)	19/626 (3.0)	**1.8** (1.2 to 2.9)
**Low-moderate** Flow rate (L/min): 3–4 FiO_2_ (%): 25–28	122/5077 (2.4)	30/558 (5.4)	**2.2** (1.5 to 3.3)
**Moderate** Flow rate (L/min): 5–9 FiO_2_ (%): 29–35	37/1436 (2.6)	21/270 (7.8)	**3.0** (1.8 to 5.1)
**High** Flow rate (L/min): 10–14 FiO_2_ (%): 36–50	41/1189 (3.4)	20/196 (10.2)	**3.0** (1.8 to 4.9)
**Very high** Flow rate (L/min): ≥15 FiO_2_ (%): ≥51	46/984 (4.7)	34/212 (16.0)	**3.4** (2.3 to 5.2)

*Data are presented as the number of observation sets triggering an escalation/total number of observation sets (%).

†Risk ratio is calculated as (risk of escalation for observation sets with rising inspired oxygen)/(risk of escalation for observation sets with stable inspired oxygen). Risk ratios that differ significantly from one are highlighted.

FiO_2_, fractional inspired oxygen concentration.

**Table 2:** Risk of escalation according to the presence or absence of individual NEWS-2 parameters scoring 3

NEWS-2	No parameters scoring 3*	At least one parameter scoring 3*	Risk ratio (95% CI)†
3	28/7104 (0.4)	7/250 (2.8)	**7.1** (3.1 to 16.1)
4	36/5216 (0.7)	16/344 (4.7)	**6.7** (3.8 to 12.0)
5	53/2341 (2.3)	49/724 (6.8)	**3.0** (2.0 to 4.4)
6	53/1263 (4.2)	64/1095 (5.8)	1.4 (0.98 to 2.0)
7	38/574 (6.6)	103/1065 (9.7)	**1.5** (1.02 to 2.1)
8	10/174 (5.7)	81/750 (10.8)	1.9 (0.99 to 3.5)
9	2/49 (4.1)	57/469 (12.2)	3.0 (0.7 to 11.8)

*Data are presented as the number of observation sets triggering an escalation/total number of observation sets (%).

†Risk ratio is calculated as (risk of escalation for observation sets with at least one parameter scoring three)/(risk of escalation for observation sets with no parameters scoring three). Risk ratios that differ significantly from one are highlighted.

NEWS, National Early Warning Score-2.

### Factors associated with a change in treatment

**Figure 2:**
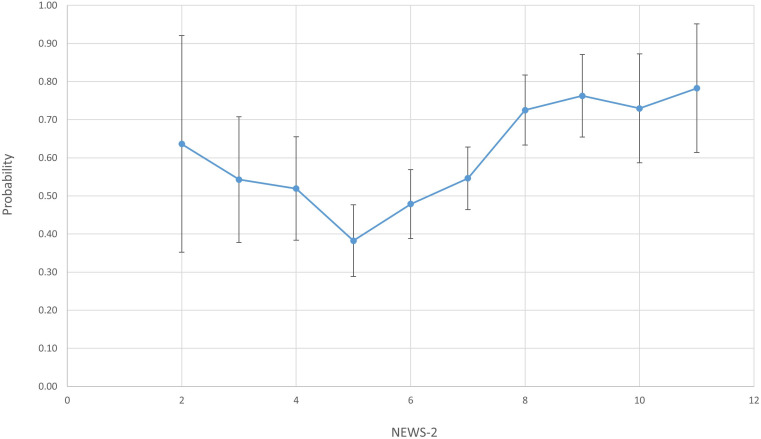
Probability that an escalation results in a change in treatment according to National Early Warning Score-2 (NEWS-2). Error bars signify 95% CIs.

Figure 2 shows that there was a U-shaped relationship between NEWS-2 and the probability of a given escalation resulting in a change in treatment. 38% of escalations with a NEWS-2 of five resulted in a change in treatment, whereas higher or lower values of NEWS-2 were associated with a higher probability of a change in treatment. Table 3 shows the percentage of escalations resulting in a change of treatment according to whether each NEWS-2 parameter was within the normal range. Individual NEWS-2 parameters did not appear to have a large influence in most cases. 83.6% of escalations with a reduced level of consciousness led to a change in treatment, compared with 55.4% in patients who were alert (p<0.001); 60.7% of escalations with a high or low respiratory rate led to a change in treatment, compared with 52.1% with a normal respiratory rate (p=0.029).

**Table 3:** Percentage of escalations resulting in a change in treatment according to normal or abnormal values of National Early Warning Score-2 parameters

	Normal	Abnormal	P value
Heart rate	116/213 (54.5)	280/474 (59.1)	0.258
Systolic blood pressure	266/445 (59.8)	130/242 (53.7)	0.125
Respiratory rate	126/242 (52.1)	270/445 (60.7)	0.029
Oxygen saturations	160/283 (56.5)	236/404 (58.4)	0.624
Inspired oxygen	71/135 (52.6)	325/552 (58.9)	0.185
Consciousness	350/632 (55.4)	46/55 (83.6)	< 0.001
Temperature	270/478 (56.5)	126/209 (60.3)	0.354

Data are presented as the number of escalations leading to a change in treatment/total number of escalations (%).

P values are from a χ^2^ test assessing the difference between proportions.

### Association between hospital mortality and escalations

In-hospital mortality was 24.4% in patients who were escalated at least once, compared with 7.3% in patients who were not escalated (p<0.001, χ^2^ test). In a logistic regression model (table 4), escalation at least once was a significant positive predictor of in-hospital mortality, independently of average NEWS-2 score.

**Table 4:** Logistic regression model of associations between hospital mortality, average NEWS-2 score and escalation

Predictor	OR for hospital mortality (95% CI)	P value
Average NEWS-2 score	2.32 (1.95 to 2.75)	<0.001
Escalation at least once	4.00 (2.42 to 6.62)	<0.001

ORs refer to a one-point increase in average NEWS-2 score, or at least one escalation compared with no escalations during the admission episode.

NEWS-2, National Early Warning Score-2.

## Discussion

### Main findings

We have described the functioning of NEWS-2 within the real-life setting of an acute respiratory unit in a large NHS Trust with a well-established rapid response system. We found that most NEWS-2 scores of ≥5 (93.5%) did not directly lead to a medical escalation. Conversely, about 15% of patients who were escalated had a NEWS-2<5. Rising oxygen requirement was strongly associated with escalation. Extreme values of a single observation tended to increase the likelihood of escalation compared with multiple mildly abnormal values. There was a U-shaped relationship between NEWS-2 score and the probability of an escalation leading to a change in treatment, with the minimum at a NEWS-2 of 5. In-hospital mortality was independently associated with having at least one urgent out-of-hours medical review.

### Clinical context

Our results are concordant with previous studies demonstrating that failure to escalate patients with physiological abnormalities is common, even in hospitals with established rapid response systems.^9–12^ A number of studies have shown associations between failure to escalate or delayed escalation and hospital mortality.^11–14^ However, this does not prove a causal link, since it is possible that some patients were not escalated because they had reached their ceiling of care. Moreover, it is accepted in national guidance that many patients can have a raised NEWS-2 in their steady state, while others may have a transiently raised NEWS-2 due to stress or exertion.^15^ Therefore, it is recommended that nursing staff use their clinical judgement to prioritise who needs to be seen first, taking account of their clinical concern about the patient and whether there has been a significant change in the NEWS-2 score. Implicit in this guidance is an acceptance that there are resource implications for the adoption of an early warning score such as NEWS-2, which can generate over 35 alerts per 100 patient-days in a typical acute hospital population.^4^ Even if these alerts are filtered by ward nurses and hospital coordinators before reaching medical staff, this still imposes a large mental burden on these staff groups, with the potential for alert fatigue and human error. We also found that the standard NEWS-2 escalation threshold of ≥5 fails to detect about 15% of episodes of physiological deterioration leading to escalation. Further studies are needed to determine the frequency of failure-to-rescue events due to inappropriate de-escalation of high NEWS-2 scores or not reaching the NEWS-2 escalation threshold.

Previous studies have found that the discrimination of NEWS can be improved by adding a measure of oxygen demand.^16 17^ We have shown that it is in fact rising oxygen demand that is most closely associated with escalations, rather than the absolute oxygen requirement per se. Prospective studies are needed to determine whether the addition of rising oxygen demand can improve the discrimination of NEWS-2.

We observed a U-shaped relationship between NEWS-2 and the probability of an escalation leading to a change in treatment. It may be that escalations with low and high NEWS-2 scores are qualitatively different, with low NEWS-2 escalations associated with significant nursing concern and high NEWS-2 escalations with severe physiological derangement—in each case with a high chance that a change in treatment will be needed. Escalations with a NEWS-2 score of exactly 5 may fall between these two categories, perhaps often being escalated due to ‘protocol’ rather than true nursing concern and not being associated with severe physiologic derangement—hence leading to a rather low chance of treatment being changed. The fact that escalations with a NEWS-2 score <5 had a relatively high chance of leading to a change in treatment highlights that physiological scoring systems cannot detect all deteriorating patients, and they cannot replace the clinical judgement of ward nurses.

We found that patients who were escalated at least once had a significantly higher in-hospital mortality than those who were not escalated, even after accounting for average NEWS-2 score. This suggests that patients who were at greatest risk of adverse outcomes were correctly identified and prioritised for medical review. Nevertheless, approximately 7% of patients who were not escalated died during their admission, leaving open the possibility that failure to rescue may have occurred in some of these cases.

The regular measurement of clinical observations has been accepted practice for a number of decades. However, the time taken to undertake these is not insignificant, and has been estimated at 5 min per observation set.^18^ In this study, a relatively low percentage of clinical observations (1.1%) resulted in a change in patient management. It is possible that alternative methods of patient monitoring such as the use of wearable devices, supplemented by machine learning-based predictive algorithms, may eventually supplant manual measurement and interpretation of clinical observations.^19^

### Limitations

The main limitation of the study was that it was restricted to data from patients admitted under the care of adult respiratory medicine in a single hospital trust. Our findings require validation in a broader cohort of medical and surgical patients, and in a variety of hospital settings, in order to ensure generalisability. A further limitation was that while we determined the proportion of observation sets which led to an escalation under different circumstances, we could not objectively determine the appropriateness or otherwise of escalation decisions. In addition, we limited our analysis to the current clinical observations, and did not specifically examine the effect of previous clinical observation sets on escalation decisions.

### Conclusions

Escalation decisions in a real-world setting are made based on a combination of early warning scores and clinical judgement. Rising oxygen requirement and extreme values of individual observations are particularly associated with the occurrence of urgent out-of-hours medical reviews, suggesting that these factors are prioritised by ward nurses and hospital coordinators when deciding whether to escalate a patient.

## Data Availability

No data are available. Current ethical and institutional approvals do not allow data sharing. Proposals for research collaboration involving data sharing should be made to the corresponding author, and will be subject to an ethics amendment and data sharing agreement.
